# The Effects of Age, Biological Maturation and Sex on the Development of Executive Functions in Adolescents

**DOI:** 10.3389/fphys.2021.703312

**Published:** 2021-09-10

**Authors:** Felien Laureys, Lotte Middelbos, Nikki Rommers, Silke De Waelle, Eline Coppens, Mireille Mostaert, Frederik J. A. Deconinck, Matthieu Lenoir

**Affiliations:** ^1^Department of Movement and Sports Sciences, Ghent University, Ghent, Belgium; ^2^Department of Movement and Sport Sciences, Vrije Universiteit Brussel, Brussels, Belgium

**Keywords:** executive function, maturation, adolescence, development, generalized estimation equation

## Abstract

The development of executive functions (EF) has been widely investigated and is associated with various domains of expertise, such as academic achievement and sports performance. Multiple factors are assumed to influence the development of EF, among them biological maturation. Currently the effect of biological maturation on EF performance is largely unexplored, in contrast to other domains like physical development or sports performance. Therefore, this study aimed (a) to explore the effect of chronological age on EF performance and (b) to investigate to what extent age-related changes found in EF are affected by biological maturation on both sexes. To this end, EF performance and degree of maturity, indexed by percentage of predicted adult height (%PAH), of 90 adolescents (11–16 years old, 54% males) were measured on three occasions in a time frame of 12 months. A Generalized Estimating Equation (GEE) approach was used to examine the association between chronological age and %PAH and the weighted sum scores for each EF component (i.e., inhibition, planning, working memory, shifting). All models were run separately for both sexes. The males’ results indicated that EF performance improved with age and degree of maturity on all four components. Interaction effects between age and %PAH on inhibition showed that at a younger age, males with a higher %PAH had a lower chance of performing well on inhibition, whereas at later ages, males with a higher %PAH had a higher chance to have a good inhibition performance. For working memory, it seems that there is no maturity effect at a younger age, while at later ages, a disadvantage for later maturing peers compared to on-time and earlier maturing male adolescents emerged. Females showed slightly different results. Here, age positively influenced EF performance, whereas maturity only influenced inhibition. Interaction effects emerged for working memory only, with opposite results from the males. At younger ages, females with lower %PAH values seem to be scoring higher, whereas at later ages, no maturity effect is observed. This study is one of the first to investigate the effect of biological maturation on EF performance, and shows that distinct components of EF are influenced by maturational status, although the effects are different in both sexes. Further research is warranted to unravel the implications for maturation-driven effects on EF that might significantly affect domains of human functioning like academic achievement and social development.

## Introduction

Executive functions (EF) are cognitive processes required for the behavioral control of numerous daily-life tasks and are crucial for cognitive, social, and psychological development (Diamond, 2013). Typically, EF performance and development are investigated from a “chronological age”-point of view. However, it is known that there is considerable inter-individual variation in the rate and timing of biological maturation, which makes chronological age an estimate of development at best (Lloyd et al., 2014). This is especially true for adolescence, which is accompanied with many biological within-person changes (Grumbach and Styne, 1998). Biological maturation refers to the timing and tempo of the progress toward the mature biological state related to growth (Malina et al., 2004). In contrast to the EF research field, studies on physical development in the sports context have widely investigated and applied the impact of biological maturation (Cumming et al., 2017). These studies generally indicate an advantage for early maturing adolescents compared to late maturing adolescents on sports performance during these pubertal phases due to their advanced growth and physical fitness level (Meylan et al., 2010; Rommers et al., 2019). Biological maturation could have a similar influence on EF and EF development (Juraska and Willing, 2017; Chaku and Hoyt, 2019; Stumper et al., 2020), and can affect academic performance, social development or even risk behavior (Magnusson et al., 1985; Baxter-Jones et al., 2005; Koerselman and Pekkarinen, 2017).

Four main EF factors are categorized during adolescence, i.e., inhibition, shifting, working memory and planning (Miyake et al., 2000; Laureys et al., 2021). Inhibition is associated with “the deliberate, controlled suppression of prepotent responses” (Miyake et al., 2000). Shifting concerns switching between multiple tasks, operations or mental sets (Miyake et al., 2000). Working memory refers to remembering, monitoring, coding incoming information and updating information (Miyake et al., 2000; Nemati et al., 2017); and planning is related to problem solving (Laureys et al., 2021). These four EF components (i.e., inhibition, planning, shifting, working memory) will further be used in this paper to determine EF performance during adolescence.

In spite of the abundance of publications on EF and its factor structure, the development of EF is not fully understood. EF development is associated with brain maturation in general and specifically with maturation of the prefrontal cortex (Diamond, 2002; Huizinga and Smidts, 2011), resulting in a relatively rapid improvement of all four EF components during childhood (until the age of 12) in comparison to early and late adolescence (12–18 years old) (Anderson et al., 2001). During adolescence (around the age of 15), the rate of improvement decreases and adult levels of EF are attained (Huizinga et al., 2006). Zooming in on the adolescent phase, the suggestion that biological maturation may influence the rate and timing of EF development has repeatedly been made (Best and Miller, 2010). Two main hypotheses have been presented to explain such effects. The hormonal influence hypothesis suggests that during adolescence, an increase in sex hormones in the brain are related to the start and end of sensitive periods in the brain that affect EF and behavior (Chaku and Hoyt, 2019; Laube and Fuhrmann, 2020). At the onset of adolescence, the release of these sex hormones can cause an excess of synapses, a phenomenon that can evoke a decrease in the quality of information processing and possibly even EF performance [see Blakemore and Choudhury (2018) for a review]. However, once the pruning process of neural pathways has started, the prefrontal cortex is also reorganized, leading to more efficient cognitive processing (Crone, 2009; Koolschijn et al., 2014; Juraska and Willing, 2017; Chaku and Hoyt, 2019; Laube and Fuhrmann, 2020).

The second hypothesis takes into account the biological influence, as well as the social changes and challenges that are associated with adolescence (Ge and Natsuaki, 2009). This maturation disparity hypothesis states that, compared to their peers, earlier maturing adolescents might encounter more physical, cognitive and social challenges they are not always emotionally ready to cope with. The hypothesis is mainly investigated in females, and only rarely in males. Although this hypothesis is often used to clarify a higher number of psychopathological symptoms in early maturers, there could also be beneficial consequences for EF (i.e., increase in attention for early maturers; Chaku and Hoyt, 2019). The small number of studies on the relationship between EF development and biological maturation contrasts with the potential impact of maturation-driven differences EF might have on an individual’s success in the social, academic, and professional domains (Diamond, 2013). From that perspective, further clarification of the association between biological maturation and EF is mandatory.

Next to differences in the timing and tempo of EF development, biological maturation could also play a role in sex-related differences during EF development. A recent review of Grissom and colleagues (2019) showed that there still is a lot of ambiguity in sex differences on the different EF components. Some studies indicate equal EF performance between males and females from childhood to adulthood (Grissom and Reyes, 2019), while in other studies females are found to outperform males on inhibition and working memory (Laureys et al., 2021). Especially during adolescence, possible sex differences in EF development could be explained by different timing and rate of biological maturation and the underlying hormonal processes (Anderson et al., 2001). Females typically mature earlier than males, where females start the adolescent period around 10–11 years, and males at around 11.5 years old (Malina and Bouchard, 1992). The difference in timing of maturation is also visible in brain maturation, more specifically, in the increase in frontal gray matter that reaches its peak at different ages for both sexes (11.0 years for females and 12.1 years for males) (Giedd, 2004).

Until now, EF development has been mainly investigated from a “chronological age”-perspective. Hence, the main goal of this study is to examine the influence of biological maturation on age-related differences in EF between 11- to 16-years-old adolescents. Because of the difference in rate and timing of biological maturation between males and females, we expect differences in EF performance. Therefore, we will investigate the influence on EF separately for males and females. The hypothesis is that older adolescents will perform better than their younger peers, and that by the end of the puberty phase, early maturing adolescents will also have an advantage over late maturing adolescents.

## Materials and Methods

### Participants

A convenience sample of 94 Flemish adolescents between 11 and 16 years participated in this study. The participants were all recruited from the first until the fifth year at one secondary school. Only data of students who participated in at least two or three test occasions were included for further analyses, leading to a total of 90 participants (54% males). From these 90 participants, 88 were present at the first test occasion, 84 at the second test occasion and 85 at the third test occasion. Reasons for drop-out were sickness at the day of testing or changing schools between test occasions.

This project has been conducted in accordance with the Helsinki Declaration and was approved by the Ethical Committee of the Ghent University Hospital (number 2017/1548). Since all participants were minors, parents or their legal representatives gave their written informed consent. All data were analyzed confidentially.

### Study Design

A mixed-longitudinal follow-up design with three test occasions was set up to measure the influence of biological maturation on EF development. Originally, it was intended to have 4 months between each test moment. The first occasion was in October 2019, the second in January-February 2020. The third test moment was planned in May 2020, but due to the COVID-19 pandemic including the closing of the schools in Flanders, this test moment was postponed until October 2020.

During each test occasion, anthropometric characteristics were measured, and EF performance was assessed using an online EF test battery. Both tests were administered in a separate room with enough space for the participants to work without disturbance of others. At least two of the researchers were present to explain all the tests separately and to answer questions. Before the first test occasion, parents were asked to complete a form with demographic data, including birth date and sex of the participant, and the biological parents’ body height.

### Measurements

#### Anthropometry

During each test moment, stature and weight of the participants were measured. Stature was measured to the nearest 0.1 cm using a portable stadiometer. The participants were asked to stand barefoot with their heels against the stadiometer and with their head in a neutral position. Weight (0.1 kg) was measured using a digital scale. Body mass index (BMI, kg/m^2^) was calculated with the height and weight measurements from the first test occasion. Further BMI classifications were made, based upon the BMI cut-off scores developed by the International Obesity Task Force (IOTF) (Cole and Lobstein, 2012). With these cut-off scores, the adolescents were categorized as being underweight, normal-weight, overweight or obese.

#### Percentage of Predicted Adult Height

The measured anthropometrics of the participants were used to determine the percentage of predicted adult height (%PAH) of each individual. In this study, the Khamis–Roche method was used (Khamis and Roche, 1995). With this Khamis–Roche method, an estimation of the biological maturation was made, based on anthropometrics. Therefore, the biological age of individuals can be biologically ahead of (early maturing), on time with (average maturing) or behind (late maturing) their chronological age. This type of estimation is previously linked to the pubertal status estimated with the Tanner stages (Cumming et al., 2017). The adolescent’s chronological age, body height and weight, as well as the body height of both biological parents, were entered in the following equation:


$$\begin{array}{c} \mathrm{Predicted adult stature}\;\left(\text{cm}\right)\\ \;=\beta_{0}+\beta_{1}\cdot \text{stature}\left(\text{cm}\right)+\beta_{2}\cdot \text{weight}\left(\text{kg}\right)\\ \;+\beta_{3}\cdot \mathrm{mid}\mathrm{parent}\mathrm{stature}\left(\mathrm{cm}\right) \end{array}$$

The intercept (β_0_) and coefficients (β_1,_ β_2,_ β_3_) in the equation depend on age and sex.

No objective classification of early, on-time, and late mature adolescents could be made in this study. Instead, we compared EF performance of earlier (same age and sex peers with a higher %PAH) and later (same age and sex peers with a lower %PAH) maturing adolescents within this sample.

#### Cambridge Brain Sciences Test

The EF test battery used in this study was the web-based Cambridge Brain Sciences (CBS) test battery. The tests used in the CBS, are all computerized versions of well-known and widely used neuropsychological tests to measure EF constructs. Test-retest reliability of the CBS test battery further proved to be decent (*r* = 0.68) (Hampshire et al., 2012). All tests were administered online, on a 9.7-inch iPad 2017 (iOS 12.1, Apple Inc., Cupertino, CA, United States).

The CBS test battery can contain up to thirteen EF tests, including a wide range of outcome variables. In this study, seven tests were used: Spatial Span, Double Trouble, Token Search, Odd One out, Spatial Planning, Monkey Ladder and Sustained Attention to Response tasks (SART). The Spatial Span is derived from the Corsi Block Tapping task (Corsi, 1972). An adapted version of the Stroop task is used here as the Double Trouble task (Stroop, 1992). The Token Search, otherwise known as the Spatial Search task, is based upon the work of Collins et al. (1998). The Odd One Out is a computerized version of the classic fluid intelligence test, used by (among others) Brenkel et al. (2017). The Spatial Planning task is an online version of the Tower of London task (Shallice, 1982). The Monkey Ladder is a visual-spatial working memory task derived from non-human primate literature (Inoue and Matsuzawa, 2007). Lastly, the SART is based upon a Go/No-Go task (Robertson et al., 1997). The Spatial Span, Token Search and Monkey Ladder are tests to assess visual-spatial working memory. To assess inhibition, the Double Trouble and Sustained Attention to Response tasks are used. The Odd One Out was included to evaluate shifting performance and the Spatial Planning for planning performance. Detailed information about the seven specific tasks and the outcome measures is included in Supplementary Material 1. These seven tests were always assessed in the same order as described above. All tests started with the same (low) level of difficulty for each participant, and the complexity increased or decreased depending on the accuracy of response.

### Data Analysis

The raw CBS scores were converted into a weighted sum score for the four EF components separately (inhibition, working memory, planning and shifting). These sum scores were calculated based on the four-factor model and factor loadings as described by Laureys et al. (2021). The outcome measure per EF task was multiplied by their respective standardized factor loading for each EF component. The sum per EF component of these weighted scores was then calculated. Detailed information about the four-factor model and factor loadings, can be found in Supplementary Material 2. Based on the weighted sum scores for each EF component, means and standard deviations (SD) could be calculated per age group (11.75–12.74; 12.75–13.74; 13.75–14.74; 15.75–16.74) and %PAH group (79–85; 86–90; 91–95; 96–100). To end up with equal ranges for both the age and %PAH group, categorization of these groups was based on the lowest and highest numbers for both variables. Means and SD were also provided for the EF components and anthropometric data per sex, age and/or %PAH group. To facilitate comparison of age- and maturity-related changes between the different EF components, the mean difference between the oldest and youngest age group, and most and least mature group across all three test occasions was expressed as a percentage for each EF component.

To examine the influence of age and biological maturation and the interaction between these factors on EF performance, a generalized linear model is necessary which investigates population average effects. Therefore, a Generalized Estimating Equation (GEE) approach (Gaussian family) was used. This approach requires repeated measures over time, without providing insight into longitudinal change over time (within individuals). Participants with at least two of the three time points completed, were included in this analysis. All individual data points (e.g., the two or three measurements of each participant are considered as single data points) were used to make population-based prediction plots, while still accounting for the non-independence (i.e., intra-personal clustering) of EF scores recorded at different time points for the same participant. Chronological age and %PAH were included in the model as continuous predictor variables and the weighted sum scores for each EF component as the outcome variable. Age and %PAH were added both separately and in interaction with each other in the different GEE models. Because differences in maturational timing for males and females occur during adolescence, the GEE models were run separately for both sexes. In total, three models were fit for each EF component and per sex, including the following independent variables:

(1) EF × Age
(2) EF × %PAH
(3) EF × (Age × %PAH)

Variance Inflation Factor (VIF) was checked as a measure of multicollinearity. If the VIF factor scores above 10, collinearity is present and interaction effects should be excluded from the analyses (Bagheri, 2015). The age and %PAH models were compared using quasi-likelihood under the independence model criterion (QICu) (Pan, 2001; Xu et al., 2019). Generally, a lower QICu indicates a better model fit. All statistical analyses were conducted in R (version 3.5.2), and STATA (version 16.1) was used to visualize the results.

## Results

An overview of the means and standard deviations for each of the four EF components per sex, age and %PAH group is provided in Table 1. To allow for qualitative comparison of age- and maturity-related differences across the four EF components, the difference between the score of the oldest and youngest age group, and most and least mature group were expressed as a percentage score. The difference between the youngest participants (11.75–12.74) and the oldest participants (15.75–16.74) was 29% for inhibition, 38% for planning, 8% for shifting and 9% for working memory. With regard to %PAH, the group with highest %PAH (96–100%) scored 15% higher on inhibition, 51% higher on planning, 14% higher on shifting and 10% higher on working memory than the group with the lowest %PAH (79–85%). Next to EF descriptive values, we also provided information about the height, weight, BMI and %PAH per sex and age group in Table 2. BMI could not be calculated for five out of 90 participants (height and/or weight was missing; 5.6%). When BMI cut-offs were used on the first test occasion, 14 participants were classified as underweight (15.6%), 64 as normal-weight (71.1%), seven as overweight (7.8%) and no adolescents were classified as obese.

**TABLE 1:** Number of participants (N) and means with standard deviation (M ± SD) of each EF component per sex and per age and %PAH.

	N	Inhibition	Planning	Shifting	Working memory
**Age**					
**11.75–12.74**	**37**	**0.52 ± 0.1**	**2.09 ± 1.1**	**15.40 ± 2.7**	**9.44 ± 1.2**
Male	12	0.53 ± 0.1	1.96 ± 1.2	14.36 ± 2.3	9.51 ± 1.5
Female	25	0.52 ± 0.1	2.14 ± 1.1	15.88 ± 2.7	9.41 ± 1.0
**12.75–13.74**	**69**	**0.51 ± 0.1**	**2.41 ± 1.3**	**15.80 ± 2.3**	**9.42 ± 1.5**
Male	37	0.51 ± 0.1	2.17 ± 1.0	15.27 ± 1.8	9.33 ± 1.7
Female	32	0.51 ± 0.1	2.68 ± 1.6	16.41 ± 2.7	9.53 ± 1.2
**13.75–14.74**	**68**	**0.56 ± 0.1**	**2.16 ± 0.9**	**15.57 ± 2.3**	**9.23 ± 1.3**
Male	43	0.56 ± 0.1	2.16 ± 0.9	15.02 ± 2.3	9.33 ± 1.4
Female	25	0.57 ± 0.1	2.17 ± 1.0	16.54 ± 2.1	9.05 ± 0.9
**14.75–15.74**	**63**	**0.60 ± 0.1**	**2.53 ± 1.1**	**15.84 ± 2.3**	**9.75 ± 1.4**
Male	37	0.59 ± 0.1	2.33 ± 1.2	15.19 ± 2.3	9.84 ± 1.4
Female	26	0.61 ± 0.1	2.82 ± 0.8	16.77 ± 1.8	9.61 ± 1.3
**15.75–16.74**	**23**	**0.67 ± 0.1**	**2.88 ± 1.2**	**16.70 ± 1.7**	**10.27 ± 1.7**
Male	14	0.68 ± 0.1	2.83 ± 1.4	16.21 ± 1.7	10.18 ± 1.9
Female	9	0.65 ± 0.1	2.97 ± 0.8	17.44 ± 1.3	10.40 ± 1.3
**% PAH**					
**79–85**	**45**	**0.55 ± 0.1**	**2.00 ± 0.8**	**14.76 ± 2.0**	**9.51 ± 1.4**
Male	36	0.55 ± 0.1	1.96 ± 0.9	14.86 ± 1.9	9.49 ± 1.5
Female	9	0.52 ± 0.1	2.14 ± 0.5	14.33 ± 2.3	9.61 ± 0.8
**86–90**	**72**	**0.51 ± 0.1**	**2.42 ± 1.3**	**15.79 ± 2.6**	**9.43 ± 1.3**
Male	40	0.51 ± 0.1	2.22 ± 1.0	15.15 ± 2.3	9.29 ± 1.4
Female	32	0.51 ± 0.1	2.67 ± 1.6	16.59 ± 2.8	9.60 ± 1.2
**91–95**	**87**	**0.59 ± 0.1**	**2.33 ± 1.1**	**16.10 ± 2.3**	**9.35 ± 1.5**
Male	46	0.61 ± 0.1	2.28 ± 1.1	15.41 ± 2.2	9.59 ± 1.7
Female	41	0.57 ± 0.1	2.39 ± 1.1	16.90 ± 2.1	9.07 ± 1.1
**96–100**	**33**	**0.63 ± 0.1**	**3.02 ± 1.0**	**16.82 ± 1.7**	**10.42 ± 1.3**
Male	9	0.68 ± 0.1	3.43 ± 1.5	16.44 ± 1.7	11.33 ± 1.4
Female	24	0.61 ± 0.1	2.86 ± 0.8	16.96 ± 1.7	10.08 ± 1.1

*Categories are made here to describe the raw data means and standard deviations for age and %PAH. %PAH, percentage of predicted adult height. Bold values are the values for male and female participants together within this category.*

**TABLE 2:** Descriptive information about height, weight, BMI and %PAH with means and standard deviation (M ± SD) per sex and age group.

Age	Height	Weight	BMI	%PAH
**11.75–12.74**	**155.5 ± 7.7**	**42.8 ± 8.3**	**17.6 ± 2.3**	**84.5 ± 2.7**
Male	153.2 ± 5.2	39.1 ± 4.8	16.7 ± 1.7	81.8 ± 1.2
Female	156.6 ± 8.5	44.5 ± 9.0	18.0 ± 2.4	85.9 ± 2.1
**12.75–13.74**	**158.3 ± 7.8**	**45.3 ± 8.6**	**18.0 ± 2.3**	**87.3 ± 3.2**
Male	156.9 ± 9.1	43.1 ± 8.7	17.4 ± 2.2	85.0 ± 2.3
Female	160.0 ± 5.6	47.98 ± 7.6	18.7 ± 2.2	90.1 ± 1.2
**13.75–14.74**	**164.4 ± 9.0**	**52.6 ± 10.3**	**19.4 ± 3.0**	**90.8 ± 2.9**
Male	165.9 ± 9.5	52.5 ± 10.7	18.9 ± 2.6	89.2 ± 2.4
Female	161.6 ± 7.2	52.8 ± 9.6	20.2 ± 3.5	93.6 ± 1.0
**14.75–15.74**	**168.9 ± 7.9**	**59.8 ± 8.9**	**21.0 ± 2.6**	**94.7 ± 2.2**
Male	171.5 ± 7.0	60.0 ± 9.2	20.4 ± 2.6	93.3 ± 2.0
Female	165.3 ± 7.5	59.7 ± 8.5	21.8 ± 2.3	96.4 ± 0.6
**15.75–16.74**	**172.3 ± 8.4**	**63.2 ± 10.7**	**21.2 ± 2.5**	**97.2 ± 1.4**
Male	174.9 ± 6.6	66.7 ± 11.3	21.7 ± 2.9	96.5 ± 1.4
Female	168.3 ± 9.3	57.4 ± 6.2	20.3 ± 1.3	98.2 ± 0.4

*BMI, body mass index; %PAH, percentage of predicted adult height. Bold values are the values for male and female participants together within this category.*

### Age

**FIGURE 1:**
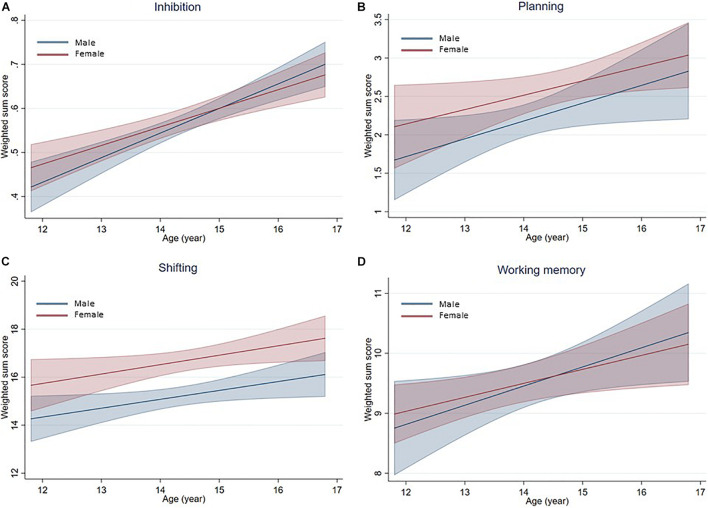
Prediction plots of the means and standard deviations based on GEE Model 1. Per EF component, a prediction of the mean with its standard deviation in relation to age is made, based upon the weighted sum scores. Blue represents males, red females. **(A)** Inhibition. **(B)** Planning. **(C)** Shifting. **(D)** Working Memory.

The first GEE models used the available data points of 49 males and 41 females, and showed a significant effect of age for both sexes, with older participants having significantly higher scores on all four components compared to their younger peers. The results are presented in Table 3 under Model 1. A visualization of this set of GEE models can be found in Figure 1. Here, we can interpret a gradual increase in performance on all four EF components for both sexes.

**TABLE 3:** Outcomes of the three GEE models split out for males and females, for each EF component with the unstandardized coefficient (b), the standard error (SE) and the 95% Confidence Interval (95% CI).

	Model 1:					Model 2:					Model 3:				
	EF × Age					EF × PAH					EF × (Age × PAH)				
EF	b	SE	95 % CI		QICu	b	SE	95 % CI		QICu		b	SE	95 % CI	
**Male**															
INH	0.05**	0.01	0.03	0.07	5.62	0.01**	0.00	0.01	0.02	5.52	age	–0.31	0.19	–0.68	0.06
											PAH	−0.06**	0.03	–0.11	–0.01
											PAH × age	0.00**	0.00	0.00	0.01
PLAN	0.24**	0.11	0.03	0.46	165.53	0.06**	0.03	0.00	0.11	156.12	age	–1.46	1.88	–5.15	2.22
											PAH	–0.25	0.30	–0.84	0.34
											PAH × age	0.02	0.02	–0.02	0.06
SHIFT	0.37**	0.17	0.03	0.71	639.36	0.08**	0.04	0.01	0.16	578.16	age	–1.52	3.45	–8.27	5.24
											PAH	–0.31	0.49	–1.28	0.66
											PAH × age	0.02	0.04	–0.05	0.09
WM	0.32**	0.15	0.03	0.60	344.88	0.08**	0.03	0.02	0.15	326.22	age	−4.31**	1.98	–8.19	–0.42
											PAH	−0.60*	0.31	–1.21	–0.01
											PAH × age	0.05**	0.02	0.01	0.09

	**b**	**SE**	**95 % CI**		**QICu**	**b**	**SE**	**95 % CI**		**QICu**		**b**	**SE**	**95 % CI**	

**Female**															
INH	0.05**	0.01	0.03	0.07	5.33	0.01**	0.00	0.01	0.02	5.33	age	–0.24	0.28	–0.79	0.30
											PAH	–0.04	0.03	–0.10	0.01
											PAH × age	0.00	0.00	0.00	0.01
PLAN	0.18**	0.09	0.01	0.35	157.83	0.04	0.03	–0.01	0.10	147.39	age	–1.16	2.57	–6.19	3.87
											PAH	–0.15	0.26	–0.67	0.36
											PAH × age	0.01	0.02	–0.03	0.06
SHIFT	0.39**	0.19	0.03	0.76	593.00	0.11*	0.06	–0.01	0.23	549.28	age	4.00	5.67	–7.12	15.11
											PAH	0.04	0.63	–0.81	1.66
											PAH × age	–0.04	0.06	–0.14	0.07
WM	0.24**	0.10	0.04	0.44	163.62	0.04	0.03	–0.02	0.09	147.60	age	–3.02	2.28	–7.49	1.46
											PAH	−0.54**	0.24	–1.01	–0.08
											PAH × age	0.04*	0.02	0.01	0.08

b, unstandardized coefficient; SE, standard error; EF, executive functions; %PAH, percentage of predicted adult height. **p ≤ 0.05 and *p < 0.1.

### %PAH

**FIGURE 2:**
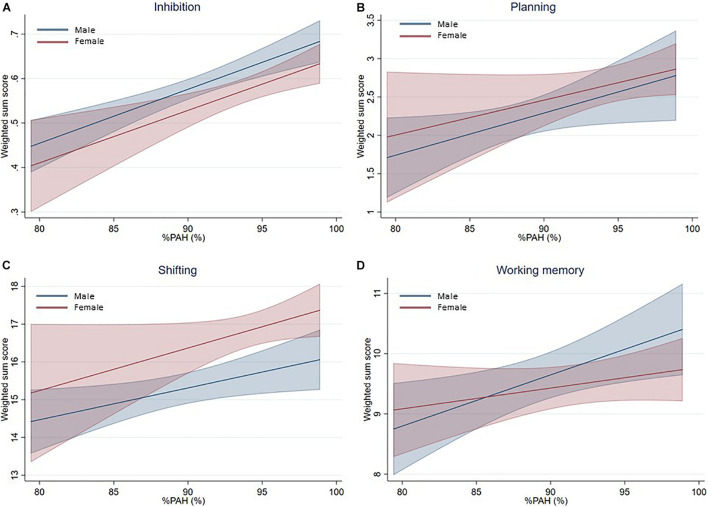
Prediction plots of the means and standard deviations based on EEG model 2. Per EF component, a prediction of the mean with its standard deviation in relation to %PAH is made, based upon the weighted sum scores. Blue represents males, red females. %PAH, percentage of predicted adult height. **(A)** Inhibition. **(B)** Planning. **(C)** Shifting. **(D)** Working Memory.

For the second set of GEE models (Model 2, Table 3), the influence of %PAH was investigated in more detail. Since %PAH could not be calculated for eight participants (i.e., body height of biological mother and/or father was missing), 45 male clusters and 37 female clusters were used in these analyses. Results from these models showed different results for both sexes. Male participants with a higher %PAH have significantly higher scores on all four EF components than participants with a lower %PAH value. In females, a higher %PAH led to higher scores on inhibition only. A marginal influence of %PAH was observed for shifting. These models are visualized in Figure 2, which again suggests that the EF performance improves with increasing %PAH, for both sexes.

### Interaction Age With %PAH

**FIGURE 3:**
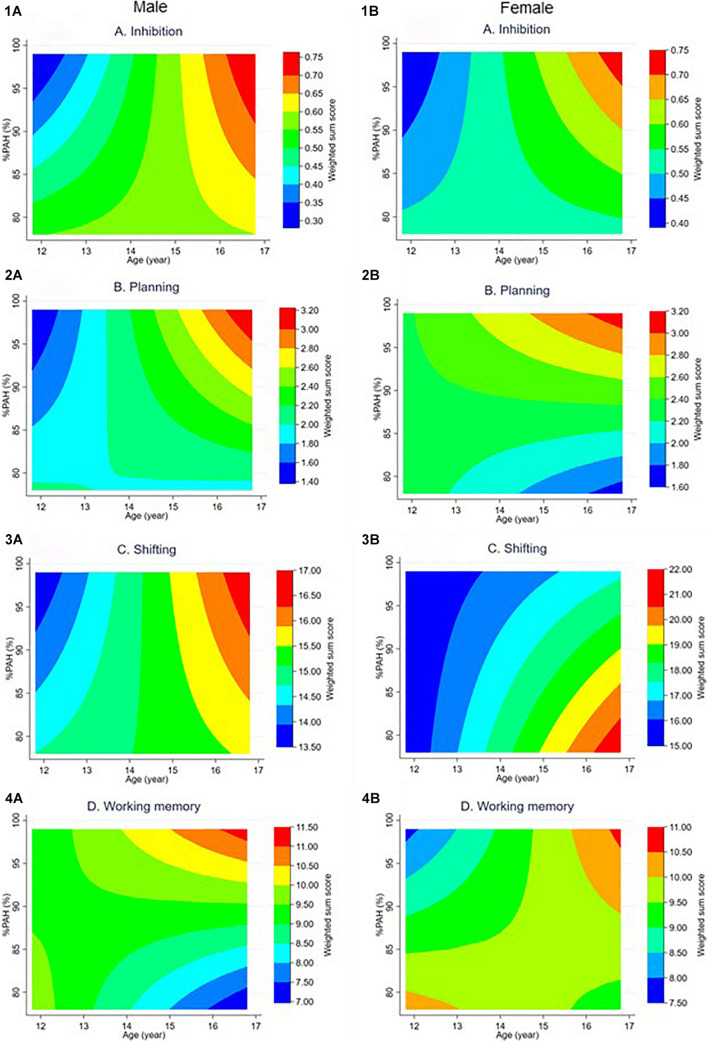
Prediction plots based on the interaction between age and %PAH in GEE model 3 per sex (A = male, B = female), for each EF component (1 = Inhibition, 2 = Planning, 3 = Shifting, 4 = Working memory). On the x-axis, age is portrayed and, on the y-axis, %PAH. EF performance is divided in seven equal intervals, going from the lowest (blue color) to highest (red color) score on the particular EF component. %PAH, percentage of predicted adult height.

Before running the interaction models between age and %PAH, the VIF score was checked. In this study, the VIF-score was 3.34, which implies no multicollinearity issues. In this final set of models (Model 3, see Table 3), 45 male clusters, 37 female clusters were used again. Main effects of age and %PAH on EF performance, addressed in Model 1 and 2, are not considered in Model 3. Results for the interaction effects of each EF component are interpreted using the prediction plots (see Figure 3).

For inhibition, the age by %PAH interaction was significant for males. The males’ prediction plot shows that, at a younger age, adolescents with a higher %PAH are likely to score lower than adolescents with lower %PAH. Around the age of 15, there seems to be a shift in this trend. Adolescents with higher %PAH are now more likely to have a better performance on inhibition than adolescents with lower %PAH. No significant interaction effect was found for females in inhibition. However, the females’ plot shows a similar pattern, with the shift at a slightly earlier age.

No significant interaction effect of age and %PAH was found for planning in either of the sexes. Nevertheless, the males’ plot for planning is similar to the inhibition plot, although higher scores (i.e., indicated by the yellow, orange, and red colors) are only seen at a later age. The female prediction plot indicates that at the younger ages, performance is rather low (indicated by the blue colors). It is only around the age of 14 that the scores gradually increase for planning. From this age onward, adolescents are more likely to perform better with age or %PAH.

For shifting again, the increase in EF performance with age is without interaction of biological maturation for both sexes. Shifting scores increase with age, independent of %PAH.

The prediction plot for working memory shows a different image, with marginal effects for both males and females. Here it seems that, until the age of 13–14 years old, all male adolescents score medium to high on the working memory task, without a clear distinction between those with a lower or a higher %PAH. Around the age of 14, more distinct effects of biological maturation emerges. From that age onward, male adolescents at the higher-end of %PAH are more likely to have a better performance. In contrast, male adolescents at the lower-end of %PAH have more chance to have lower scores for working memory. The females’ working memory plot shows a different pattern. Younger female adolescents with a higher %PAH seem to score lower on working memory. However, from 14 years old and onward, a high working memory score is observed, independent of the adolescents’ %PAH.

## Discussion

The current study explored the association of age, biological maturation and sex on EF development during adolescence. For males, age and %PAH separately are positively associated with EF performance on all four EF components (inhibition, working memory, planning and shifting). Furthermore, a significant interaction between age and %PAH was observed for inhibition and working memory, indicating that the effect of maturity varied across age. For shifting and planning, no interaction between age and %PAH was found. Age also positively influenced all four components for females, whereas maturity only influenced inhibition. The age and %PAH interaction effect only emerged for working memory, showing that females at younger ages and with lower %PAH values seem to be scoring higher, whereas at later ages, the score is similar for females with higher and lower %PAH.

The results of the current study indicate that EF performance improves with chronological age. Although a small percentage of the increase could potentially be attributed to practice effects, results are in line with previous research, indicating that EF keep developing during adolescence, although at a lower rate than during childhood (Anderson et al., 2001; Best and Miller, 2010). We observed variation in the overall % difference scores in all EF components. For shifting and working memory, relatively low differences of only 8 and 9% were observed between the oldest and youngest age group. Although other studies found a plateau for shifting performance around late childhood (12 years old) (Huizinga et al., 2006; Best and Miller, 2010), we still observed a small increase in score per year indicated by the GEE model next to the age-related differences. The relatively low level of complexity of the shifting and working memory tasks might explain this variation. More complex EF tasks are indeed documented to keep increasing at higher rates and at later ages (Miyake et al., 2000; Huizinga et al., 2006). In our study, the inhibition and planning components are based on these more complex tasks (i.e., an adapted version of the Stroop task and Tower of London task), and we also observed a difference in performance of 29% and 38% between the oldest and youngest group of adolescents.

Mixed results are observed for the influence of biological maturation in both sexes. Biological maturation significantly influences all four EF components for males and seems to explain less variance in females. Nevertheless, when the age and %PAH models are compared, a better model fit (i.e., indicated by lower QICu values) is seen for %PAH across all EF components and both sexes. There are several possible explanations for the differences between both sexes. One could be that the females in this study already passed the onset of puberty and were systematically further down their maturation process compared to the males, since higher female %PAH values were seen. Therefore, it could be that the females in this study already passed the sensitive period for changes in plasticity caused by hormonal and neural reactions. Secondly, when observing the standard deviations on the average %PAH, the variation is smaller for females than males. Although this could also indicate that females indeed are reaching the end of maturity before the males, it might also indicate that there is more variation in timing of maturity in males. When the majority of females are “on-time” maturers, it is hard to detect differences in timing of maturity, possibly explaining why biological maturation has less influence on EF performance here. In general, the different results on EF performance might indicate that the existence of sex-related differences on EF performance could not only be related to chronological age, but also to biological maturation. We suggest that future studies expand the sample size in both sexes and start measuring both biological maturation and EF performance already at a younger age (around 10 years old) up until older ages (around 18 years old), to examine this potential mechanism in more detail.

Biological maturation did not influence age-related differences in performance on shifting and planning for both sexes, but a significant interaction effect between age and %PAH was found for inhibition (males) and working memory (males and females). More specifically for the males, earlier maturing adolescents scored lower on inhibition compared to on-time and later mature adolescents, at a younger age. However, the difference seems to reduce during adolescence, and eventually, the earlier mature male adolescents outperform their later maturing peers at later ages on inhibition. For working memory, average scores were observed for young male adolescents, independent of maturity. By the end of the adolescent period, the later maturing adolescents are more likely to have a lower performance score, compared to the average to very good performance for earlier and on-time maturing adolescents. Female adolescents have slightly different results for working memory. At a younger age, earlier maturing adolescents are having lower scores, when females are older high working memory scores are observed independent of biological maturation. On both inhibition and working memory, a temporarily lower score is seen for earlier maturing adolescents. This could possibly be due to the reorganization of the prefrontal cortex, induced by sex hormones (Chaku and Hoyt, 2019; Laube and Fuhrmann, 2020), although the temporary decline in performance should eventually emerge in all adolescents at some chronological age, when puberty onset starts, a general temporary decline in score was not observed in this study. The maturation disparity hypothesis can explain why the short decrease in scores could potentially only happen to earlier maturing adolescents, because they might encounter more challenges at puberty onset than their peers, resulting in different EF scores during adolescence (Ge and Natsuaki, 2009; Laube and Fuhrmann, 2020). We should also take into account that females have a 2-year head start in their biological maturation process compared to males (Malina et al., 2004). This might explain why for working memory, an influence of biological maturation was found at the older ages for males, but not for females. The end of the adolescent period in females occurs around the age of 16–17. Since the adolescent period for males can last until 18–19 years old, it could be that similar results can be found at these ages. Detailed neuroimaging studies are required to fully comprehend the brain maturation process during adolescence for both sexes and shed light on the possible differences between early and late maturing adolescents on EF development.

The results of the current study using GEE models are promising to measure the influence of the timing of biological maturation on EF performance. Nevertheless, some limitations and future recommendations should be addressed. First, future studies should benefit by expanding the age range. Both younger age groups (closer to onset of puberty for females) and older age groups (closer to end of puberty for males) could be included to clarify the influence of biological maturation on EF performance and perhaps also indicate when the plateau of EF performance begins. Second, in the current study GEE models predict the influence of biological maturation, based on the raw scores of the individual data points of all participants. However, the majority of the participants are more likely to be maturing on-time, with only a few earlier and later maturing adolescents (compared to their same-age, same-sex peers within this sample), as is seen in a normal population. It should be noted that with each method estimation biological maturation (e.g., Khamis–Roche method), some degree of error should be taken into account. Furthermore, prediction for participants at the extreme ends of the maturation continuum (i.e., earlier or later maturing adolescents) could have a larger error rate and should be interpreted with caution. Future studies could benefit from a longer follow-up period with a wider age range and a larger sample size to investigate the interaction between EF development and biological maturation in more detail. Third, the current study used seven tasks of the CBS test battery, each with specific performance indicators. Since more research is revealing that the EF factor structure is in part dependent on the selected tasks and outcome measures (Karr et al., 2018), it would be advisable to replicate the current study with the seven EF tasks used in this study and perhaps add other EF tasks to see if the same results will hold up. Nevertheless, and in contrast with many underpowered research concerning EF factor structure, the performance indicators resulted in a four-factor structure with weighted sum scores was based on a large sample (>2,000 participants), as was established by Laureys et al. (2021). Lastly, other influential factors, such as socioeconomic status (Jacobsen et al., 2017) IQ (Ardila et al., 2000) or physical activity (Alghadir et al., 2019) could be included to further examine their role in EF performance during adolescence.

This study examined the influence of biological maturation on EF performance during adolescence. Previous research traditionally analyzed EF development in function of chronological age, but our results indicate that biological maturation should also be taken into account, and even provides a better fit when examining EF performance. This is especially the case in research where maturation could potentially clarify differences in EF development and sex differences on EF performance. However, it is also important in daily-life settings, since EF can affect academic, social, and emotional development during adolescence. The pattern of interaction between age and biological maturation differs between EF components and between both sexes, probably related to maturational timing. Inhibition and working memory are clearly affected by the timing and tempo of biological maturation, while the effect on planning and shifting was minimal.

## Data Availability Statement

The data will be made available upon reasonable request to the corresponding author.

## Ethics Statement

The studies involving human participants were reviewed and approved by Ethical Committee of the Ghent University Hospital. Written informed consent to participate in this study was provided by the participants’ legal guardian/next of kin.

## Author Contributions

FL, LM, SD, NR, EC, MM, FD, and ML were involved in the conceptualization of the study and wrote the manuscript. FL, LM, and MM were involved in data collection. FL, LM, NR, FD, and ML were involved in data analysis. All authors contributed to and approved the final version of the manuscript.

## Conflict of Interest

The authors declare that the research was conducted in the absence of any commercial or financial relationships that could be construed as a potential conflict of interest.

## Publisher’s Note

All claims expressed in this article are solely those of the authors and do not necessarily represent those of their affiliated organizations, or those of the publisher, the editors and the reviewers. Any product that may be evaluated in this article, or claim that may be made by its manufacturer, is not guaranteed or endorsed by the publisher.

## Supplementary Material

Supplementary Material 1 A detailed overview of the seven CBS tests, used in this study, with the outcome measures where the weighted sum scores for each EF components is based on, and a screenshot of each test (Figure A).

Supplementary Material 2 Additional detail on the model upon which the weighted sum scores for the four executive functions were based, as well as how this weighted sum scores were calculated.

## References

[B1] Alghadir A. H. Gabr S. A. Iqbal Z. A. Al-Eisa E. (2019). Association of physical activity, vitamin E levels, and total antioxidant capacity with academic performance and executive functions of adolescents. *BMC Pediatrics* 19:1–8. 31101100 10.1186/s12887-019-1528-1 PMC6524246

[B2] Anderson V. Anderson P. Northam E. Jacobs R. Catroppa C. (2001). Development of executive functions through late childhood and adolescence in an Australian sample. *Dev. Neuropsychol.* 20 385–406. 10.1207/s15326942dn2001_5 11827095

[B3] Ardila A. Pineda D. Rosselli M. (2000). Correlation between intelligence test scores and executive function measures [article]. *Arch. Clin. Neuropsychol.* 15 31–36. 10.1016/s0887-6177(98)00159-0 14590565

[B4] Bagheri A. (2015). Sample size impacts on high leverage collinearity-enhancing observations [article]. *Econ. Comput. Econo. Cybernetics Stud. Res.* 49 161–171.

[B5] Baxter-Jones A. D. G. Eisenmann J. C. Sherar L. B. (2005). Controlling for maturation in pediatric exercise science [article; proceedings paper]. *Pediatric Exerc. Sci.* 17 18–30. 10.1123/pes.17.1.18

[B6] Best J. R. Miller P. H. (2010). A developmental perspective on executive function. *Child Dev.* 81 1641–1660. 10.1111/j.1467-8624.2010.01499.x 21077853 PMC3058827

[B7] Blakemore S. J. Choudhury S. (2018). Development of the adolescent brain: implications for executive function and social cognition [meeting abstract]. *Eur. Neuropsychophar.* 28 S1. 10.1016/j.euroneuro.2017.12.017 16492261

[B8] Brenkel M. Shulman K. Hazan E. Herrmann N. Owen A. M. (2017). Assessing capacity in the elderly: comparing the MoCA with a novel computerized battery of executive function. *Dementia Geriat. Cogn. Dis. Extra* 7 249–256. 10.1159/000478008 28868068 PMC5567119

[B9] Chaku N. Hoyt L. T. (2019). Developmental trajectories of executive functioning and puberty in boys and girls [article]. *J. Youth Adolescence* 48 1365–1378. 10.1007/s10964-019-01021-2 30989473

[B10] Cole T. J. Lobstein T. (2012). Extended international (IOTF) body mass index cut-offs for thinness, overweight and obesity [article]. *Pediatric Obesity* 7 284–294. 10.1111/j.2047-6310.2012.00064.x 22715120

[B11] Collins P. Roberts A. Dias R. Everitt B. Robbins T. (1998). Perseveration and strategy in a novel spatial self-ordered sequencing task for nonhuman primates: effects of excitotoxic lesions and dopamine depletions of the prefrontal cortex. *J. Cogn. Neurosci.* 10 332–354. 10.1162/089892998562771 9869708

[B12] Corsi P. (1972). *Memory and The Medial Temporal Region of the Brain Ph. D, Thesis.* Montreal, QB: McGill University.

[B13] Crone E. A. (2009). Executive functions in adolescence: inferences from brain and behavior [review]. *Dev. Sci.* 12 825–830. 10.1111/j.1467-7687.2009.00918.x 19840037

[B14] Cumming S. P. Lloyd R. S. Oliver J. L. Eisennnann J. C. Malina R. M. (2017). Bio-banding in sport: applications to competition, talent identification, and strength and conditioning of youth athletes [article]. *Strength Condit. J.* 39 34–47. 10.1519/ssc.0000000000000281

[B15] Diamond A. (2002). “Normal development of prefrontal cortex from birth to young adulthood: cognitive functions, anatomy, and biochemistry,” in *Principles of Frontal Lobe Function*, eds Stuss D. T. Knight R. T. (Oxford University Press), 466–503. 10.1093/acprof:oso/9780195134971.003.0029

[B16] Diamond A. (2013). Executive functions. *Ann. Rev. Psychol.* 64 135–168. 23020641 10.1146/annurev-psych-113011-143750 PMC4084861

[B17] Ge X. J. Natsuaki M. N. (2009). In search of explanations for early pubertal timing effects on developmental psychopathology [article]. *Curr. Direct. Psychol. Sci.* 18 327–331. 10.1111/j.1467-8721.2009.01661.x

[B18] Giedd J. N. (2004). Structural magnetic resonance imaging of the adolescent brain. *Ann. N. Y. Acad. Sci.* 1021 77–85. 15251877 10.1196/annals.1308.009

[B19] Grissom N. M. Reyes T. M. (2019). Let’s call the whole thing off: evaluating gender and sex differences in executive function (vol 44, pg 86, 2019) [correction]. *Neuropsychopharmacology* 44 1344–1344. 10.1038/s41386-019-0367-y 30143781 PMC6235899

[B20] Grumbach M. M. Styne D. M. (1998). Puberty: ontogeny, neuroendocrinology, physiology, and disorders. *Williams Textbook Endocrinol.* 9:e1625.

[B21] Hampshire A. Highfield R. R. Parkin B. L. Owen A. M. (2012). Fractionating human intelligence. *Neuron* 76 1225–1237. 10.1016/j.neuron.2012.06.022 23259956

[B22] Huizinga M. Dolan C. van der Molen M. (2006). Age-related change in executive function: developmental trends and a latent variable analysis. *Neuropsychologia* 44 2017–2036. 10.1016/j.neuropsychologia.2006.01.010 16527316

[B23] Huizinga M. Smidts D. P. (2011). Age-related changes in executive function: a normative study with the dutch version of the behavior rating inventory of executive function (BRIEF) [article]. *Child Neuropsychol.* 17 51–66. 10.1080/09297049.2010.509715 21218296

[B24] Inoue S. Matsuzawa T. (2007). Working memory of numerals in chimpanzees. *Curr. Biol.* 17 R1004–R1005. 18054758 10.1016/j.cub.2007.10.027

[B25] Jacobsen G. M. de Mello C. M. Kochhann R. Fonseca R. P. (2017). Executive functions in school-age children: influence of age, gender, school type and parental education [article]. *Appl. Cogn. Psychol.* 31 404–413. 10.1002/acp.3338

[B26] Juraska J. M. Willing J. (2017). Pubertal onset as a critical transition for neural development and cognition [review]. *Brain Res.* 1654 87–94. 10.1016/j.brainres.2016.04.012 27060769 PMC5053848

[B27] Karr J. E. Areshenkoff C. N. Rast P. Hofer S. M. Iverson G. L. Garcia-Barrera M. A. (2018). The unity and diversity of executive functions: a systematic review and re-analysis of latent variable studies [review]. *Psychol. Bull.* 144 1147–1185. 10.1037/bul0000160 30080055 PMC6197939

[B28] Khamis H. J. Roche A. F. (1995). Predicting adult stature without using skeletal agethe khamis-roche method (vol 94, pg 504, 1994) [correction, addition]. *Pediatrics* 95 457–457. 7936860

[B29] Koerselman K. Pekkarinen T. (2017). *The Timing of Puberty and Gender Differences in Educational Achievement.* Bonn. Available online at: https://ssrn.com/abstract=3010667

[B30] Koolschijn P. Peper J. S. Crone E. A. (2014). The influence of sex steroids on structural brain maturation in adolescence [article]. *PLoS One* 9:e83929. 10.1371/journal.pone.0083929 24416184 PMC3885531

[B31] Laube C. Fuhrmann D. (2020). Is early good or bad? Early puberty onset and its consequences for learning. *Curr. Opin. Behav. Sci.* 36 150–156. 10.1016/j.cobeha.2020.10.005

[B32] Laureys F. De Waelle S. Barendse M. Deconinck F. J. A. Lenoir M. (2021). A developmental perspective on the nature of executive functioning from childhood to adolescence. *Manus. Subm. Publi.*

[B33] Lloyd R. S. Oliver J. L. Faigenbaum A. D. Myer G. D. De Ste Croix M. B. A. (2014). Chronological age vs. biological maturation: implications for exercise programming in youth [review]. *J. Strength Condit. Res.* 28 1454–1464. 10.1519/jsc.0000000000000391 24476778

[B34] Magnusson D. Stattin H. Allen V. L. (1985). Biological maturation and social development: a longitudinal study of some adjustment processes from mid-adolescence to adulthood. *J. Youth Adoles.* 14 267–283. 10.1007/bf02089234 24301221

[B35] Malina R. Bouchard C. (1992). *Growth, Maturation, and Physical Activity.* Philadelphia, PA: LWW.

[B36] Malina R. M. Bouchard C. Bar-Or O. (2004). *Growth, Maturation, and Physical Activity.* Champaign, IL: Human Kinetics.

[B37] Meylan C. Cronin J. Oliver J. Hughes M. (2010). Talent identification in soccer: the role of maturity status on physical, physiological and technical characteristics [review]. *Int. J. Sports Sci. Coaching* 5 571–592. 10.1260/1747-9541.5.4.571 29106335

[B38] Miyake A. Friedman N. P. Emerson M. J. Witzki A. H. Howerter A. Wager T. D. (2000). The unity and diversity of executive functions and their contributions to complex “frontal lobe” tasks: a latent variable analysis [article; proceedings paper]. *Cogn. Psychol.* 41 49–100. 10.1006/cogp.1999.0734 10945922

[B39] Nemati P. Schmid J. Soltanlou M. Krimly J.-T. Nuerk H.-C. Gawrilow C. (2017). *Planning and Self-Control, But Not Working Memory, Directly Predict Multiplication Performance In Adults.* Trier: PsychOpen GOLD.

[B40] Pan W. (2001). Akaike’s information criterion in generalized estimating equations. *Biometrics* 57 120–125. 10.1111/j.0006-341X.2001.00120.x 11252586

[B41] Robertson I. H. Ridgeway V. Greenfield E. Parr A. (1997). Motor recovery after stroke depends on intact sustained attention: a 2-year follow-up study. *Neuropsychology* 11:290. 10.1037/0894-4105.11.2.290 9110335

[B42] Rommers N. Mostaert M. Goossens L. Vaeyens R. Witvrouw E. Lenoir M. (2019). Age and maturity related differences in motor coordination among male elite youth soccer players [article]. *J. Sports Sci.* 37 196–203. 10.1080/02640414.2018.1488454 29913097

[B43] Shallice T. (1982). Specific impairments of planning. *Philos. Trans. R. Soc. London B Biol. Sci.* 298 199–209. 10.1098/rstb.1982.0082 6125971

[B44] Stroop J. R. (1992). Studies of interference in serial verbal reactions. *J. Exp. Psychol. General* 121:15. 10.1037/0096-3445.121.1.15

[B45] Stumper A. Mac Giollabhui N. Abramson L. Y. Alloy L. B. (2020). Early pubertal timing mediates the association between low socioeconomic status and poor attention and executive functioning in a diverse community sample of adolescents [article]. *J. Youth Adoles.* 49 1420–1432. 10.1007/s10964-020-01198-x 32020488 PMC7302968

[B46] Xu C. Li Z. Xue Y. Zhang L. J. Wang M. (2019). An R package for model fitting, model selection and the simulation for longitudinal data with dropout missingness [article]. *Commun. Statist. Simul. Comput.* 48 2812–2829. 10.1080/03610918.2018.1468457 32346220 PMC7188076

