# Supplementary material for: The Effects of Age, Biological Maturation and Sex on the Development of Executive Functions in Adolescents
Source: Front Physiol. 2021 Sep 10;12:703312. doi: 10.3389/fphys.2021.703312 (PMC8461056; doi:10.3389/fphys.2021.703312)
Supplement: Supplementary Material 1 — A detailed overview of the seven CBS tests, used in this study, with the outcome measures where the weighted sum scores for each EF components is based on, and a screenshot of each test (Figure A). [file Data_Sheet_1.zip › Supplementary_Material_1.docx]

Supplementary Material 1:

A detailed overview of the seven CBS tests, used in this study, with the outcome measures where the weighted sum scores for each EF components is based on, and a screenshot of each test (Figure A). Test-retest reliability scores per test were added (Hampshire et al; Robertson et al.)

*Spatial Span (SS)* is a task based on the Corsi Block Tapping Task (Corsi, 1972) and measures a persons’ ability to remember the relations between objects in space (r = 0.62). This test consists of a grid of 4x4 boxes, that will light up in a random order on the screen. Participants were instructed to tap the boxes in the same sequence as they previously appeared on the screen. The first trial always had a span length of four blocks. When a trial was executed correctly (correct locations in the correct order) the next trial contained one extra box. An incorrect trial was followed with a trial containing one box less. The test ended after three incorrect responses. Response accuracy (SS RA) was used as performance indicator for the spatial span task, and was calculated as the maximum number of blocks remembered correctly for each participant.

*Double Trouble (DT)* is an adaption of the Stroop test and mainly assesses inhibitory control (Stroop, 1992). Three words are presented to the participant and participants were asked to indicate which of two coloured words at the bottom described the colour of the word at the top (r = 0.92). The test lasted 90 seconds in which participants had to give as many correct responses as possible. For this test, three performance indicators were selected. First, total response accuracy (DT RA) was calculated as percentage of correct trials for each participant. Second, mean response time (i.e. the time between the words appearing on screen and the participants tapping on a word) on double incongruent trials (DT RT II) was calculated for each participant. Double incongruent trials were trials where the top word and target word were different and had a different colour. Third, mean response time on double congruent trials (DT RT CC) was calculated for each participant. Double congruent trials were trials where both top word and target word were the same and had the same colour.

*Token Search (TS)* is a self-guided search task that mainly assesses spatial working memory (Collins et al., 1998). Participants were presented with a number of boxes randomly placed on the screen and were asked to find a token that was hidden underneath the boxes (r = 0.66). Each box contained the token only once and the next hiding place was unpredictable. The task requires to hold the selected boxes in memory. Selection of an empty box twice or a box that had previously held the token, resulted in a failure. When a trial was executed correctly (all tokens found without error) the next trial contained one extra box. After an incorrect trial the next trial contained one box less. The test ended after three incorrect responses. Response accuracy (TS RA) was selected as performance indicator for the token search task and was calculated as the maximum number of boxes found without error for each participant.

*Odd One Out (OO)* is a modern adaptation of classical tests of fluid intelligence (Brenkel et al., 2017), and mainly assesses deductive reasoning and shifting. This task consists of nine sets of shapes that differ from each other in colour, shape and size (r = 0.73). The participant had to point out which shape was the most different from the others. A correct response resulted in the next trial being more complex, while an incorrect trial would result in the next trial being less complex. The grade of complexity depended on the amount of variance on the three levels (colour, shape, size) within the nine figures. The test lasted 180 seconds in which participants had to give as many correct responses as possible. Response accuracy as well as response time were selected as performance indicators for this task. Response accuracy for the odd one out task (OO RA) was calculated as the number of correct attempts for each participant (*N attempts – N errors*). For response time (i.e. time between the trial appearing on screen and the participants tapping on a shape), the mean response time per trial was calculated for each participant (OO RT).

*Spatial Planning* (SP) is an adapted version of the Tower of London Task (Shallice, 1982), which is primarily used to assess planning ability. Participants were asked to sort balls that are positioned on a tree-shaped frame in numerical order in as few moves as possible, by replacing one ball per move (r = 0.87). The problems became progressively more complex to solve as the participant progressed through the task. The test lasted 180 seconds in which participants had to solve as many problems as possible. Response accuracy was used as a performance indicator for this task and was calculated in two steps. First, trial scores were calculated per trial using the following formula: (*minimum moves required * 2) – moves made*. The total response accuracy (SP RA) was then calculated as the sum of all trial scores for each participant.

*Monkey Ladder (ML)* is based on a task from the non-human primate literature (Inoue & Matsuzawa, 2007) and mainly assesses visuospatial working memory, or the ability to hold information in memory and to manipulate or update it depending of the purpose or the circumstances. Participants were presented with a number of boxes randomly placed on the screen, with each box containing a number ranging from 1 to the number of boxes (r = 0.57). Participants were asked to memorize the numbers appearing in each box and to tap the boxes in numerical order as soon as the numbers disappeared. When a trial was executed correctly, the next trial contained one extra box. After an incorrect trial the next trial contained one box less. The test ended after three incorrect responses. Response accuracy (ML RA) was selected as performance indicator for the monkey task and was calculated as the maximum number of boxes remembered correctly for each participant.

*Sustained Attention to Response Task* (SART, (Robertson et al., 1997) mainly assesses inhibition. Participants were presented with single digits in the centre of the screen, each digit appeared for 250 ms (r = 0.76). Participants were asked to respond with a tap on the “GO” button on the screen to each digit (GO) as quickly as possible. However, when the digit “3” appeared on screen (NO GO), participants were asked to withhold a response. Participants had to maintain their attention to this task for four minutes. The response accuracy score (SART RA NG) was calculated as the percentage of correct NO GO trials for each participant.


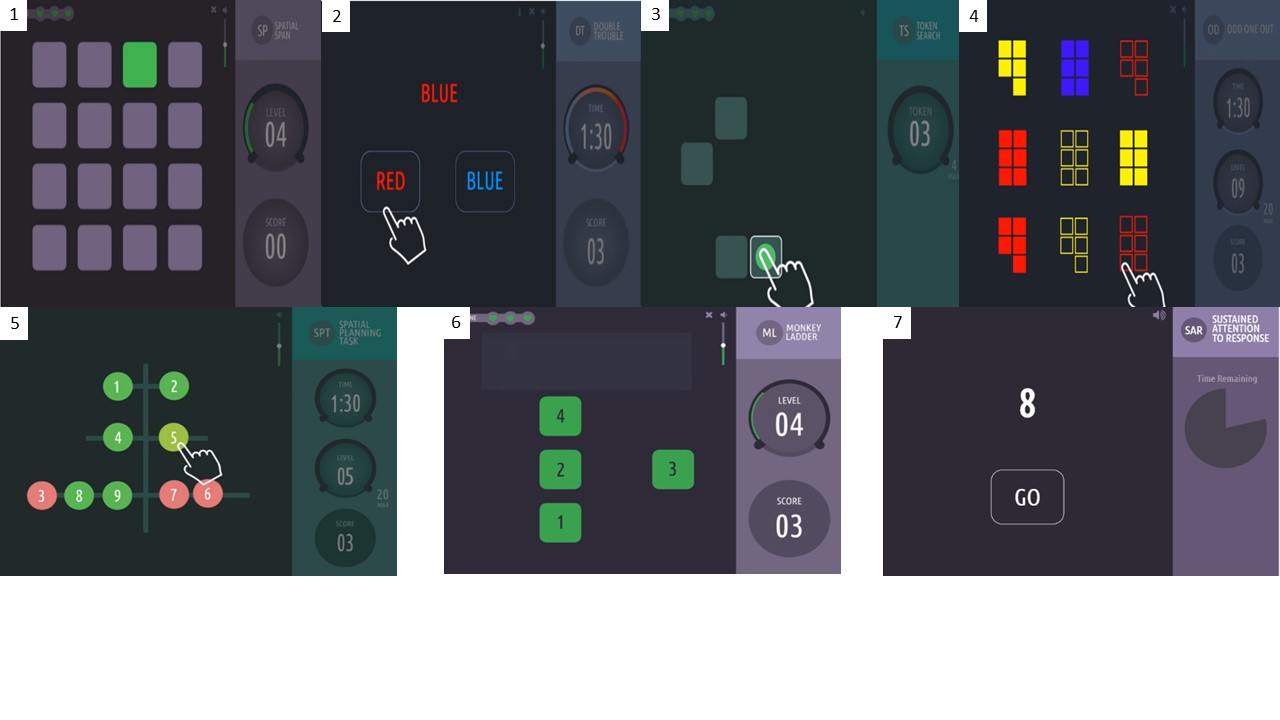


Figure A. Screenshot of the seven CBS tests. 1) Spatial Span, 2) Double Trouble, 3) Token Search, 4) Odd One Out, 5) Spatial Planning, 6) Monkey Ladder, 7) Sustained Attention to Response (SART)
