# Supplementary material for: The Effects of Age, Biological Maturation and Sex on the Development of Executive Functions in Adolescents
Source: Front Physiol. 2021 Sep 10;12:703312. doi: 10.3389/fphys.2021.703312 (PMC8461056; doi:10.3389/fphys.2021.703312)
Supplement: Supplementary Material 1 — A detailed overview of the seven CBS tests, used in this study, with the outcome measures where the weighted sum scores for each EF components is based on, and a screenshot of each test (Figure A). [file Data_Sheet_1.zip › Supplementary_Material_2.docx]

Supplementary Material 2

This appendix provides additional detail on the model upon which the weighted sum scores for the four executive functions were based, as well as how this weighted sum scores were calculated.

In a recent study by Laureys et al. (Submitted for publication), a confirmatory factor analyses using the same seven tests from this study was performed on a sample of 818 children between 12 and 17.99 years old. The results demonstrated that a four-factor model provided the best fit for this age group with these seven tests (Figure B).


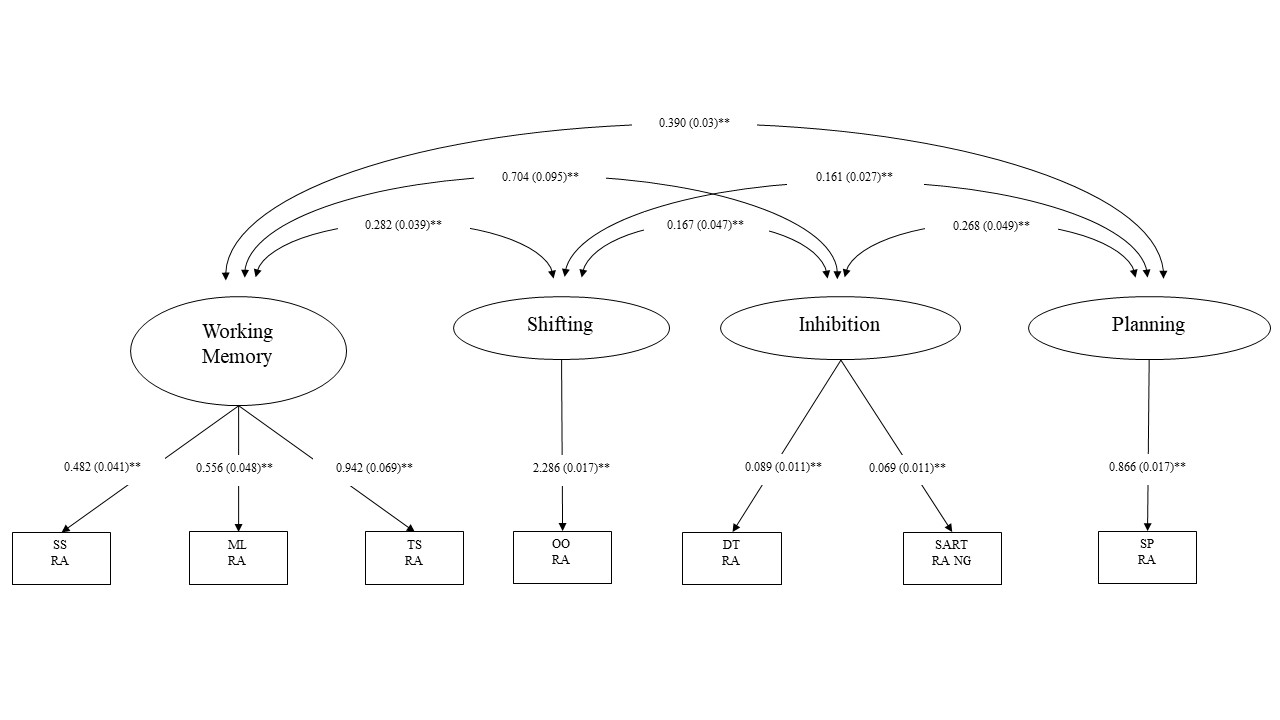


Figure B. Factor structure for the adolescent group. Estimates (standard errors) are displayed (**p < 0.001, * p < 0.05), error variances and residuals are not displayed. SS RA = Spatial Span Response Accuracy; ML RA = Monkey Ladder Response Accuracy; TS RA = Token Search Response Accuracy; OO RA = Odd One Out Response Accuracy; DT RA = Double Trouble Response Accuracy; SART RA NG = Sustained Attention To Response Task Response Accuracy No Go; SP RA = Spatial Planning Response Accuracy. (from Laureys et al., submitted for publication)

This four-factor model also includes standardized loadings for each test to evaluate the relative contribution of each test towards the four EF components, while taking into account the other tests. While the sample in the study of Laureys and colleagues was quite large, and hence allowed this kind of elaborate factor analysis, the sample of the current study was not large enough to do so. Since the sample of the study and Laureys and colleagues (submitted for publication) is representative for the Flemish youth, and thus the sample of the current study, factor loadings from the study of Laureys and colleagues could be used to calculate a weighted sum score for the four EF components, which best approaches the factor scores that would have been obtained within the original model. Hence, each individual test score was multiplied by their respective standardized factor loading for each EF factor, and then the sum of these weighted scores was calculated. Table A provides an overview of the calculated weighted sum scores with the standardized factor loading for each test.

| Table A. Overview of the calculation of the weighted sum scores with the standardized factor loadings for each test. | |
| --- | --- |
| Inhibition | = 0.572*DTRA + 0.266*SARTRANG |
| Planning | = SPRA |
| Shifting | = OORA |
| Working Memory | = 0.441*MLRA + 0.457*SSRA + 0.518*TSRA |
| *DTRA: Double Trouble Response Accuracy; SARTRANG: Sustained Attention to Response Response Accuracy No Go condition; SPRA: Spatial Planning Response Accuracy; OORA: Odd One Out Response Accuracy; MLRA: Monkey Ladder Response Accuracy; SSRA: Spatial Span Response Accuracy; TSRA: Token Search Response Accuracy* | |
